# MAGEA1 and hTERT Peptide Treatment Improves the Potency of The Dendritic Cell- Cytotoxic T Lymphocytes (DC-CTL) Immunotherapy in DAC Treated Acute Myeloid Leukemia

**DOI:** 10.7150/jca.66501

**Published:** 2022-01-24

**Authors:** Guocheng Zhong, Weiqiang Zhao, Yisheng Li, Guangyi Jin, Wei Zeng, Changhua Yu, Ji Zhou, Li Yu

**Affiliations:** 1 Department of Hematology and Oncology, Shenzhen University General Hospital, Shenzhen University Clinical Medical Academy, Shenzhen University; Shenzhen Key Laboratory, Hematology Institution of Shenzhen University, Shenzhen 518055, China; 2 International Cancer Center, Health Science Center, Shenzhen University, Shenzhen 518037, China; 3 Shenzhen Haoshi Biotechnology Co, Ltd. Shenzhen 518000, China

**Keywords:** acute myeloid leukemia (AML), dendritic cell (DC), cytotoxic T lymphocyte(CTL)

## Abstract

**Background:** Acute myeloid leukemia (AML) is a type of heterogenous malignant hematological disorder. Recently developed immunotherapies such as chimeric antigen receptor T cell (CAR-T) do not demonstrated promising therapeutic results due to the off-target effect. The Dendritic cell-cytotoxic T lymphocyte adoptive immunotherapy (DC-CTL) is one of the recently developed immunotherapies. One of the reasons that DC-CTL does not work well in AML is the lack of antigens with high binding affinity, high antigen presentation potency, and the specificity to AML cells.

**Methods:** DAC was used to treat AML cells to find overexpressed CTAs upon DAC treatment. The overexpression was confirmed at both mRNA and protein level by realtime PCR and western blotting. Peptides was designed by using the NetMHCpan database and EPIP based on the out-screened protein sequences. The peptides were then used to pulse DC-CTL coculture *in vitro* and tested the cytotoxicity of CTLs in vitro and their cancer inhibition potency *in vivo*.

**Results:** Two cancer testis antigen (CTA) proteins, MAGEA1 and hTERT, was up-regulated in DAC treated AML cells. DC cells pulsed by the antigen peptides designed based on the sequence of these two proteins demonstrated increased potency to stimulate CTL cells in terms of cytokines secretion. These cytokines included IFN-γ, IL-6, and TNF-α. Moreover, enhanced *in vitro* cytotoxicity was found in CTL cells treated with peptide pulsed DC cells. AML progress was inhibited by CTA peptides pulsed DC-CTL in a mouse AML model.

**Conclusions:** MAGEA1 and hTERT could possibly serve as specific tumor antigens upon DAC treatment, providing potential targets for the development of immunotherapies for AML in the future.

## Introduction

Acute myeloid leukemia (AML) is a type of malignant hematological disease that are phenotypically and genetically heterogenous 1. It brings a substantial economic burden to patient's family as well as the public health system due to its high mortality rate and the high medical cost 1, 2. The incidence rate of AML is about 2.25/100,000, representing 80-90% of the adult acute leukemia^3^-^4^. Various treatment strategies including chemotherapy combined with stem cell transplantation has long been used to treat AML, but the 5 -year survival rate has not been dramatically improved for the last 30 years 5, 6. Therefore, it remains imperative to develop new therapeutic methods to treat AML.

Recently, the application of chimeric antigen receptor T cell (CAR-T) therapy targeting CD19 hugely improved the therapeutic outcome for B cell derived malignant tumors, providing a new possibility to develop therapies for AML 7, 8. However, study results demonstrated a huge off-target effect of CAR-T therapies targeting CD33, CD123 in AML 9, emphasizing the urgent needs to develop highly specific antigen- based immunotherapies for AML.

Various immunotherapy strategies have been designed to treat various types of cancers, such as increasing the immunogenicity of cancer cells, and enhancing the host immune response to target cancer cells. The dendritic cell-cytotoxic T cell (DC-CTL) adoptive immunotherapy is one of such recently developed immunotherapies 10, 11. However, DC-T cell-based adoptive immunotherapy does not work effectively in AML. One of the possibly reasons may be the lack of specific and effective antigens for AML cells.

DNA methylation is one of the characteristics of AML cells. It plays an important role during AML tumorigenesis, development, and prognosis 12. Decitabine (DAC), also known as 4-Amino-1-(2-deoxy-beta-D-erythro-pentofuranosyl)-1,3,5-triazin-2(1H)-one, has been proved to inhibit the activity of DNA methyltransferases (DNMTs) to reverse the gene silence caused by high methylation level of DNA in tumor cells, therefore inhibiting the progress of cancer development 13. Meanwhile, DAC could also increase the expression of cancer testis antigens (CTAs) and the expression of major histocompatibility complex (MHC) in cancer cells 14. CTA is a group of unique tumors associated antigens that show no expression or extremely low expression in normal tissues except in testis, DAC treatment could not induce their expression in normal cells either^15^, ^16^. However, it has been reported that administration of DAC up-regulates the expression of tumor associated antigens and/or immune response associated molecules to increase the immunogenicity of tumor cells 17. In our previous work, we also found that DAC treatment enhanced the expression of CTAs in AML cells. Based on the unique expression pattern of CTAs in AML cells, it could be possibly be ideal targeting candidates of immunotherapies for AML.

Dendritic cells (DC) and T cells, especially CD8+ T cells, are the two key players during immunotherapies for tumors 18,19. DC cells are the only type of antigen presenting cells (APC) that activate naïve T cells. Its functional status determined the types and the potency of immune response. Tumor specific antigen pulsed DC cells could induce the activation and proliferation of cytotoxic T lymphocyte (CTL) to execute their anti-tumor functions. The tumor antigen pulsed DC-CTL immunotherapies have been proved effective in melanoma, renal cancers, and ovarian cancers 20, 21. Previously investigated tumor antigens included tumor cell lysate, RNA or cDNA, and antigen peptides. After being processed by DC cells, these antigens were presented to cell surface as the peptide-MHC complex (pMHC) in DC cells to activate naïve T cells. Therefore, the specificity and the immunogenicity of presented antigens are pivotal to the specific and the potency of the immune response induced by antigen-pulsed DC cells.

In the present study, we found that the expression of melanoma-associated antigen 1 (MAGEA1) and telomerase reverse transcriptase (hTERT) was elevated upon DAC treatment in NB4 cells. MAGEA1 belongs to MAGE family antigents and it has been consistently found expressed in various cancers and germinal cells 22. hTERT is known for its key role in the telomearase complex. Overexpression of hTERT is not rare in different types of cancers 23. iPeptides derived from MAGEA1 and hTERT were screened according to their binding affinity and an epitope presentation prediction software and were used to pulse DC cells. It was found that T cells activated by peptide-pulsed DC cells demonstrated higher level of cytokine secretion and increased ability to induce AML cell death compared to DAC treatment to AML cells alone and scrambled peptides. In an AML mouse model, injection of T cells activated by MAGEA and hTERT peptide-pulsed DC cells inhibited AML tumors development compared to DAC treatment alone and scrambled peptide pulsing.

## Materials and methods

### Mice and cell lines

The Balb/c mice used in this study were purchased from Guangdong Medical Laboratory Animal Center (Guangdong, China) and were maintained under pathogen-free conditions in the animal facility. All procedures involving mice were approved by the Institutional Animal Care and Use Committee of Shenzhen University and General Hospital of Shenzhen University, and all experiments were done in accordance with the US Department of Health and Human Services Guide for the Care and Use of Laboratory Animals and institutional guidelines. The human promyolocytic leukemia cell line, NB4, was cultured in RPMI 1640 medium (Thermo Fisher Scientific, Shanghai, China) supplemented with 10% fetal bovine serum (Thermo fisher Scientific, Shanghai, China), 50 IU/ml penicillin, 50μg/ml streptomycin, 2mM l-glutamine and 70 mg/L sodium bicarbonate. The cells were incubated at 37°C incubator supplemented with 5% CO_2_.

### DAC treatment of AML cells

Decitabine (DAC, Xian-Janssen pharmaceuticals Ltd, China) was dissolved in phosphate-buffered saline (PBS) (pH 7.4) to obtain 100 µM stocks and stored at -20℃. NB4 cells were cultured to logarithmic growth phase and were treated with 1 μmol/L DAC for 3 days.

### RNA sequencing and real time PCR

Trizol (Thermo Fisher Scientific, USA) was used to extract total RNA after DAC treatment, and the extracted total RNA was sent to Gene Denovo Biotechnologies (Guangzhou, China) for RNA-seq. total RNA extracted from DAC untreated NB4 cells was used as control.

Trizol extracted total RNA was also used to perform real time PCR to confirm the expression pattern of selected genes, MAGEA1 and hTERT. TaqMan Fast Advanced Master Mix (Thermo Fisher Scientific, USA) was used to do the real time PCR according to manufacturer's instruction. Primers used for real time PCR were:

MAGEA1: Forward 5'-tacctggagtaccggcaggt-3'

Reverse 5'-ttggaccccacaggaact ca-3';

hTERT: Forward5'-atgcgacagttcgtggctca-3'

Reverse5'-atcccctggcactggacgta-3';

GAPDH: Forward 5'-gaaggtgaaggtcggagtc-3'

Reverse 5'-gaagatggtgatgggatttc-3'.

### SDS-PAGE electrophoresis, mass spectrometry and western blot

5X10^6^ DAC treated and untreated NB4 cells were lysed in 300 μl RIPA buffer. After 10 minutes lysing on ice, cell lysate was centrifuge at 12000 rpm for 15 minutes. And the supernatant was removed to a new tube. Equal amount of proteins from each sample was used to run a 4-20% SDS-PAGE gel.

For Mass spectrometry, the SDS-PAGE gel was stained by Coomassie Blue, and the bands of proteins overexpressed DAC treated cells were cut and sent to do mass spectrometry for protein identity.

For western blot, equal amount of total proteins from DAC treated and untreated NB4 cells were separated by SDS-PAGE electrophoresis, transferred to PVDF membrane and perform western blot following routine procedure. MAGEA1 monoclonal antibody was purchased from Santa Cruz Biotechnologies (Shanghai, China). hTERT rabbit monoclonal antibody was purchased from Abcam (Cambridge, USA). Polyclonal GAPDH antibody was purchased from Santa Cruz Biotechnologies (Shanghai, China).

### Screening of CTA peptide candidates

Full length of amino acid sequence of MAGEA1 and hTERT were input to NetMHCpan database (http://www.cbs.dtu.dk/services/NetMHCpan-2.2/). Antigen epitope was set to HLA-A*1101 compatible, output was set to 9-11 mer, DC presentable peptides. The resulted peptides were evaluated by both: 1) EPIP (Epitope Presentation Integrated Prediction) software (Genoimmune Therapeutics, Wuhan, China) for its high presentation potency with the threshold set to ≥0.5. And 2) NetMHCpan database for its high binding affinity to MHC(HLA-A*1101), with the threshold set to ≤100. Four peptide sequences were finally selected for each protein, and the peptides were synthesized by GeneScript Biotechnologies (Nanjing, China). Peptide with randomly scrambled sequence (Sp) was also synthesized as control. The amino acid sequence of Sp peptide is: VFSTVPPAFI. The selected peptide sequence for MAGEA1 and hTERT are listed in **Table 1**.

### Isolation of human DC cells and T cells, establishment of DC-CTL coculture system

20 ml peripheral blood from a healthy volunteer was collected and provided by Shenzhen Blood Station, and an equal amount of physiological saline solution was added to the blood sample and mixed well. Ficoll-Paque Plus medium (GE, USA) was then added into the sample, and the blood sample was centrifuged at 2000 rpm for 20 minutes at room temperature. After centrifugation, the white-membrane layer (mono-nuclear cells layer) was carefully removed to a culture dish with RPMI-1640 medium and cultured in CO_2_ incubator for 2 hours at 37℃. The culture medium containing suspension cells was removed and saved for the isolation of CTL cells. The attached cells were washed with RPMI-1640 and further cultured in the fresh medium with rhGM-CSF and rhIL-4 (1000 U/ml and 500 U/ml respectively, Schering-Plough, Kenilworth, USA). rhGM-CSF and rhIL-4 were re-added to the culture medium on day 3 and day 5. At day 5, cells were stimulated with CTAp or Sp peptide at 10 ug/ml in the culture medium. For each target protein, MAGEA1 or hTERT, the 4 selected peptides were freshly prepared in the culture medium and mixed at the same ratio in mass before use. 24 hours later, TNF-α (20ng/ml, PeproTech, USA) was added to the medium to promote the maturation of DC cells for another 24 hours. At the end of day 7 after the DC cell maturation, DC cells were treated with mitomycin C for 30 minutes followed by washing and refreshing the culture medium.

The suspension cells from the saved medium were counted and further cultured in IL-2 containing medium for 7days. At the end of 7 days culture, the suspension cells were counted and cocultured with above-prepared DC cells at the ratio of DC:CTL=1: 10 for additional 7 days. Cell culture supernatant were then collected, the CD8+ T cells were isolated by MojoSort™ Human CD8 T Cell Isolation Kit (Biolegend,USA) according to manufacturer' instruction. Cleared supernatant was used to measure cytokine secretion by ELISA. Enriched CD8+ T cells were further tested for cytotoxicity *in vitro* or injected into AML mice model.

### ELISA

The secreted cytokines including IFN-γ, IL-6, and TNF-α were measured in the coculture supernatant. ELISA kits detecting human IFN-γ, IL-6 and IFN-γ (Biolegend, USA) were used to evaluate the cytokine secretion of CTL cells treated with Sp-DC or CTAp-DC cells following manufacturer's instruction.

### LDH cytotoxicity test

NB4 cells were seeded in 96 well plates and treated with DAC at 1 μmol/L DAC for 3 days. DAC untreated cells were used as control. Enriched CD8+ T cells from Sp-DC-CTL coculture or CTAp-DC-CTL coculture supernatant were counted and seeded in the DAC treated or untreated NB4 cell well at a ratio of CTL: NB4=20:1. After incubation, cell culture medium was collected, centrifuge, and the clear supernatant was used to test the cytotoxicity of CTL cells with a LDH cytotoxicity assay kit from Abcam (Cambridge, MA, USA) according to the manufacturer's instruction.

### Establishment of AML model in mice

4-5 weeks old Balb/c mice were injected with 100mg/kg bodyweight Cyclophosphamide (CTX), ip. Four days later, 5×10^6^ NB4 cells were injected via tail vein. Seven days later, peripheral blood was obtained via tail cutting to confirm the AML cell implantation by Wright-Giemsa staining.

Successfully implanted mice were randomly divided into 4 groups: Sp-DC-CTL treatment, DAC+Sp-DC-CTL treatment, CTAp-DC-CTL treatment, and DAC+Sp-DC-CTL treatment. Each group had 6 mice. Mice in DAC treatment group were injected intraperitoneally with DAC solution at 1mg/kg body weight for 3 consecutive days, once a day. Mice in non-DAC treated group were injected with same amount of sterilized water as control. Five days after the last dose of DAC treatment, 1×10^6^ Sp-DC-CTL or CTAp-DC-CTL cells were tail-vein injected into the mice according to the treatment strategy for their group. 10 days later, peripheral blood sample was collected by tail tip cutting from each mouse. Mice then sacrificed by cervical neck dislocation, spleen and bone marrow from tibias and femurs were collected to evaluate the tumor burden. Spleen size was measured by spleen weight/body weight. Peripheral blood and bone marrow smear was evaluated by Wright-Giemsa staining.

### Statistics

GraphPad software was used to analyze the data. Student t-test was used to evaluate the significance of difference among groups. Data was presented as Mean±SEM. A P-value <0.05 was considered statistically significant.

## Results

### DAC treatment increased the expression of MAGEA1 and hTERT in AML cells

**Figure 1:**
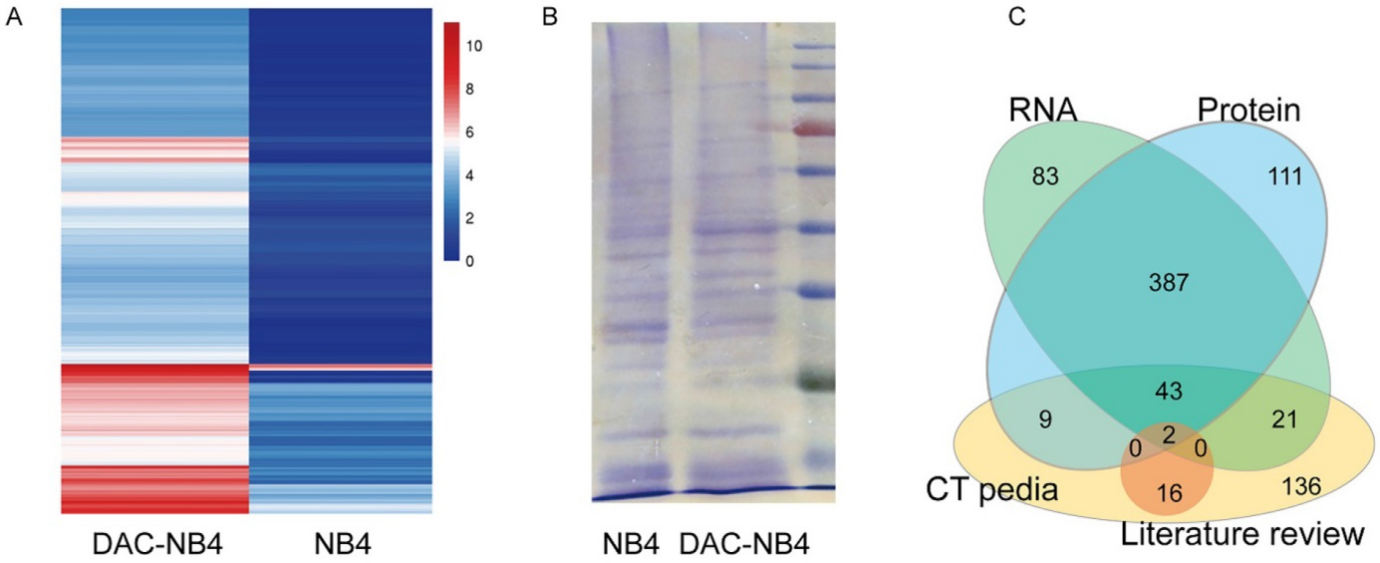
** differentially expressed proteins in NB4 cells upon DAC treatment.** NB4 cells were treated with or without DAC and the expression profile was analyzed at both mRNA level by RNA sequencing (**A**) and mass spectrometry (**B**). **A** is a heat map showing the expression profile patterns in DAC treated (DAC-NB4) and DAC untreated (NB4) cells. **B** is a representative SDS-PAGE electrophoresis result of proteins harvested from DAC treated and untreated NB4 cells. The gel was stained by Coomassie Blue. **C**. is a schematic figure showing the screening of candidate CTA proteins.

MHC class I comprised a vast variety of alleles. In China, HLA-A*1101 is the most common allele, representing 22 to 29% of the whole population. Thus, NB4 cells, a promyelocytic leukemia cell line that possesses HLA-A*1101 allele, was used to in our study. As shown in Figure 1, compared to untreated NB4 cells, DAC treated NB cells demonstrated a completely different expression pattern at mRNA level (**Figure 1 A**). 491 proteins were found up-regulated in DAC treated cells. Protein expression was also compared in DAC treated and untreated cells. Equal amount of total cell lysate was run on an SDS-PAGE gel to separate proteins according to molecular weight. Protein bands showing increased expression pattern were cut and sent to sequence for protein identification. A representative gel running image was shown in **Figure 1 B**. Proteomic analysis revealed that 507 proteins were up-regulated in DAC treated cells. We then compared the results from RNA sequencing and the proteomic analysis, as well as the available CTA in CTA database CTpedia (http://www.cta.lncc.br), those CTA proteins that were up-regulated by DAC treatment in NB4 cells at both mRNA and protein levels were picked out, resulting in 45 proteins. These 45 proteins were also compared to the currently known CTAs that are expressed in AML cells according to published literatures in databases including Pubmed, SicenceDirect, and Science Citation Index. Two proteins, MAGEA1 and hTERT, were finally screened out.

### MAGEA1 and hTERT was increased in DAC treated NB4 cells

**Figure 2:**
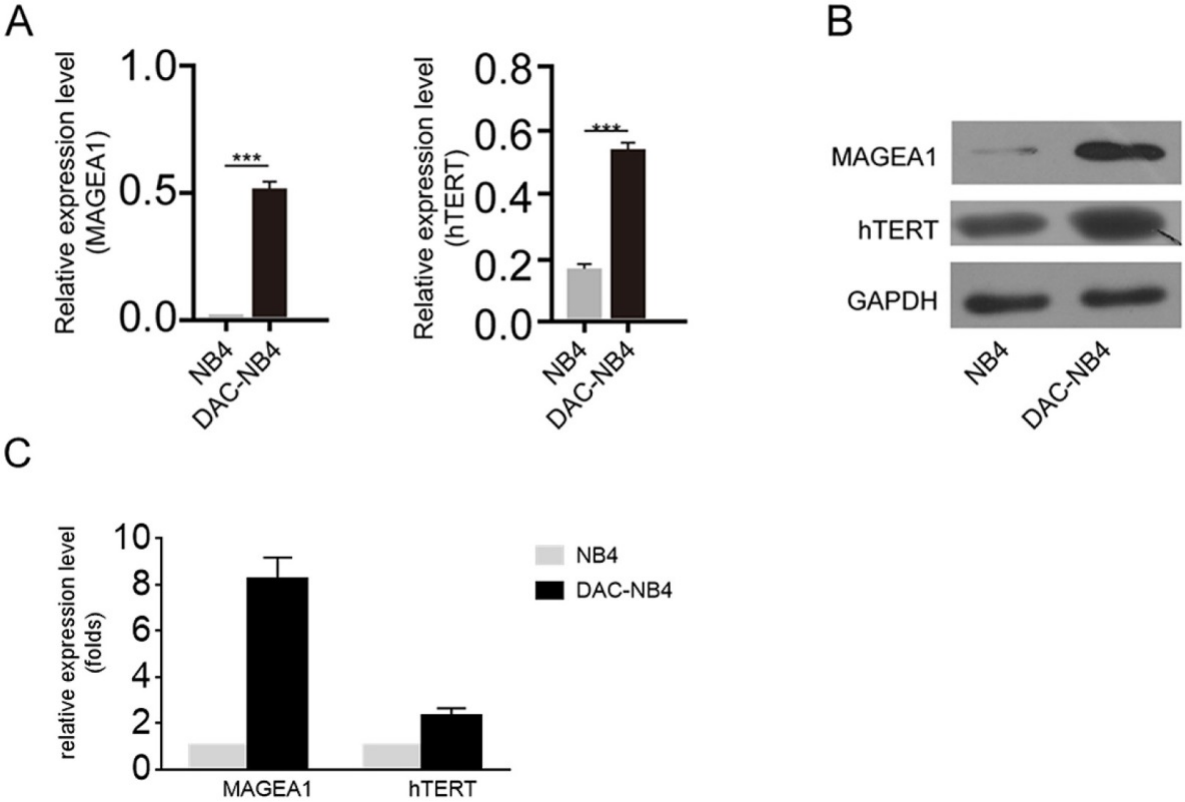
** MAGEA1 and hTERT expression was up-regulated upon DAC treatment at mRNA and protein level. A.** Quantitative real-time PCR was used to determine the expression level of MAGEA1 and hTERT upon DAC treatment. Each sample was triplicated, and the experiment was performed independently for 3 times. GAPDH was used as internal control. The difference was analyzed by student T-test. ***: P<0.001**. B.** a representative figure of western blot result for MAGEA1 and hTERT expression upon DAC treatment. GAPDH was used as internal control. The experiment was repeated at least 3 times independently.** C.** statictical analysis result of MAGEA1 and hTERT expression from 3 independent western blots.

The real time PCR and western blot were used to further confirm the overexpression of MAGEA1 and hTERT in DAC treated AML cells. As shown in Figure 2 A, after being normalized by the expression of GAPDH, MAGEA1 expression and hTERT expression were hugely elevated in DAC treated cells (**Figure 2 A**). Similar result was also obtained at protein level by western blot (**Figure 2 B, Figure 2 C**).

### Screening of MHC high-affinity, high presenting potency antigen peptides

Multiple database and software were used to screen for antigen peptides of MAGEA1 and hTERT that possess high affinity to MHC and high potency of antigen presenting. First, NetMHCpan database (http://www.cbs.dtu.dk/services/NetMHCpan-2.2) was used to predict the MHC-matched, 9-11 mer peptides derived from the DC cell processing of selected CTA, named CTAp. Resulted CTAp was evaluated by the NetMHCpan database for its MHC affinity. EPIP (epitope presentation integrated prediction) software was used to evaluate the potency of each CTAp to be presented by DC. Four peptides for each CTA (MAGEA1 and hTERT) were selected for further investigation. The sequence of each selected peptide was listed in **Table 1**.

### Cytotoxic T lymphocytes were activated by peptide pulsed DC cells *in vitro*

**Figure 3:**
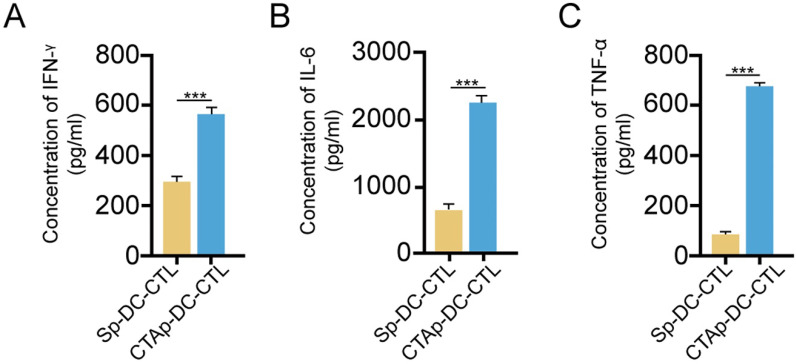
** cytokine secretion by CTL upon peptide pulsed DC stimulation.** CTL cells were cocultured with indicated peptide pulse DC cells, and the secreted cytokines by CTL cells were measured by ELISA. **A**. IFN-γ; **B**, IL-6; **C**. TNF-α. The result was analyzed with student T-test. ***: P<0.001. Sp-DC-CTL: CTL cells cocultured with Sp peptide pulsed DC cells, CTAp-DC-CTL: CTL cells cocultured with CTA peptide mixture pulsed DC cells.

Selected peptides were synthesized and mixed at equal amount, labeled as CTAp. A peptide with random scrambled sequence was used as a negative control, labeled as Sp. Human DC cells and CTL cells were isolated from peripheral blood drawn from a healthy volunteer. Five days after isolation and culture, DC cells were pulsed with CTAp in addition to TNFα. Same amount of Sp peptide was used as control. After 48 days of pulsing of DC cells by the CTAp or Sp peptides, DC cells were co-cultured with CTL cells for 7 days. At the end of 7 days of co-culture, cytokines including IFN-γ, IL-6, and TNF-α were measured in the supernatant. As shown in **Figure 3**, secretion of cytokines was dramatically increased in the co-culture of CTAp pulsed DC-CTLs compared to co-culture of Sp peptides pulsed DC-CTL, suggesting the enhanced activation of CTL cells.

**Figure 4:**
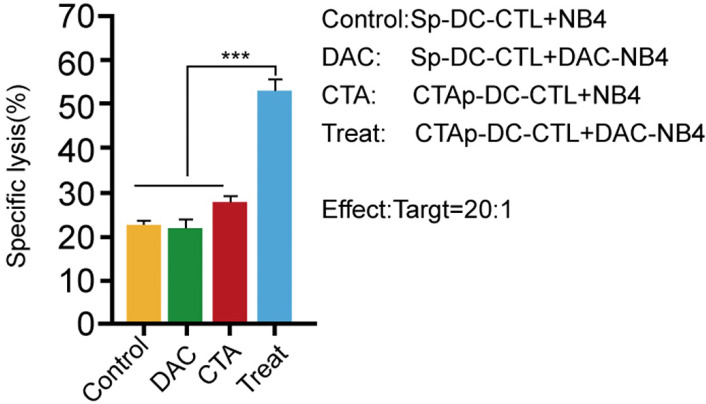
** cytotoxicity of CTL cells was enhanced by coculture with CTAa-DC cells.** Cytotoxicity of CTL cells was measured by LDH release assay kit. Each sample was triplicated, and the result was repeated at least 3 times. The result was analyzed with Student T-test. ***: P<0.001.

We tested the cytotoxicity of CTAp -DC-CTL cells. T cells in the DC-CTL co-culture were enriched by CD8+ magnetic beads, and then cocultured with DAC treated or untreated NB4 cells. Specific NB4 cell lysis was measured by LDH release. As shown in **Figure 4**, Sp-DC-CTL did not increase cells lysis in DAC treated NB4 cells compared to that in DAC untreated NB4 cells. CTAp-DC-CTL treatment only slightly, if not at all, increase the cell lysis compared to Sp-DC-CTL in DAC untreated cells, possibly due to the low expression of MAGEA1 and hTERT without DAC treatment. However, when CTAp-DC-CTL was used to coculture with DAC treated NB4 cells, NB4 cell lysis was dramatically compared to all other tested treatments, suggesting that CTAg-DC-CTL were more potent with respect to cytotoxicity.

### CTAp-DC-CTL inhibited AML in vivo

**Figure 5:**
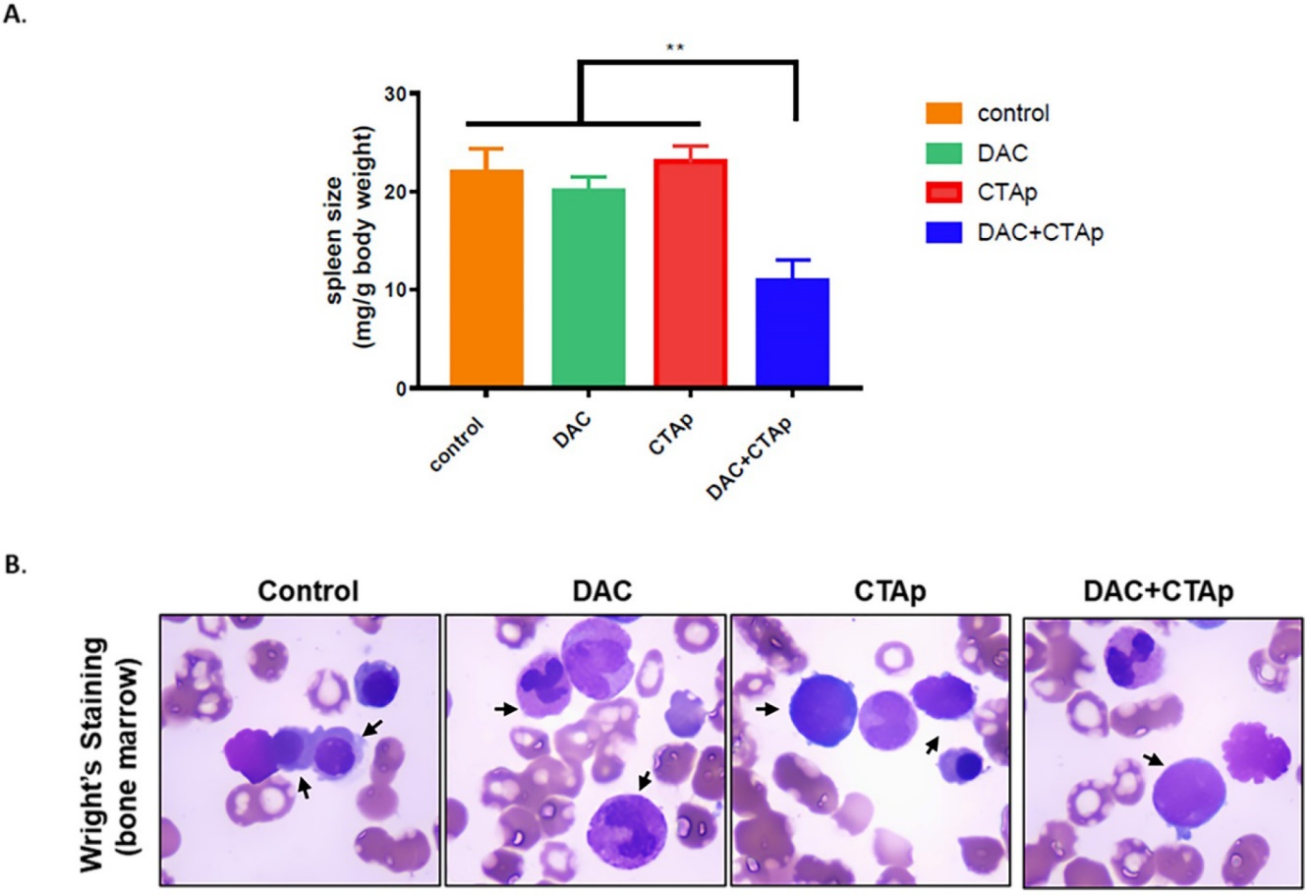
** CTAp-DC-CTL inhibited AML *in vivo.*** DC cell stimulated CTL cells were used to treat DAC treated or untreated mice implanted with AML. After treatment, spleen size, bone marrow of the mice were collected to evaluate the effect of CTAa-DC-CTL+DAC treatment.** A**. spleen size was measured by spleen weight/body weight and the result was analyzed by using student's Test. **:P<0.01.** B.** representative picture of Wright-Giemsa staining of AML cells in bone marrow after indicated treatment.

We also tested the effect of CTAp-DC-CTL in an in vivo model. NB4 cells were implanted to Balb/c mice via tail vein injection. After confirmation of successful implantation of NB4 cells by Giemsa staining of peripheral blood smear, mice were divided into 4 groups and were treated with Sp-DC-CTL treatment (Control), Sp-DC-CTL+DAC treatment (DAC), CTAp-DC-CTL treatment (CTAp), and CTAp-DC-CTL+DAC (treatment) treatment, respectively. The result was shown in **Figure 5**. At the end of the treatment, spleen size of each group of mice was measured. As shown in **Figure 5 A**, compared to control treatment, DAC treatment alone, or CTAp treatment alone, combination of DAC treatment and CTAp-DC-CTL treatment reduced the spleen size of the leukemic mice by about 50%. Bone marrow sample from each group of mice were also stained with Wright-Giemsa to check the AML cells, and the result was shown in **Figure 5 B**. Similarly, CTAp-DC-CTL combined with DAC treatment greatly reduced the number of AML cells in bone marrow compared to control treatment, DAC treatment, or CTAp-DC-CTL treatment, suggesting the great enhancement of the potency of the immunotherapy.

## Discussion

In the present study, we found that DAC treatment could increase the expression of MAGEA1 and hTERT, two CTA proteins, in a AML cell line, NB4. Antigen peptides with MHC high-affinity and high antigen presenting potency derived from these two proteins were screened out by bioinformatic approaches. By coculturing with the CTAp-pulsed DC cells, CTL cells activity and cytotoxicity were greatly enhanced *in vitro* and *in vivo*.

CTAs are promising as good candidates for cancer immunotherapy targets due to its very restricted expression pattern in normal tissues, which is very low expression or no expression except only in adult testis and in placenta but expressed in various tumor cells. As a member of the MAGE-A antigens, the best characterized CTA gene family, MAGEA1 is strictly tumor-specific and is detected in various solid tumors 24. MAGEA 3/A6 was also found up-regulated in AML cells from DAC treated AML patients 25. In our study, NB4 cell line, instead of AML patient samples were used to screen CTAs, that may explain why MAGEA1, instead of MAGEA3/A6, was detected. As a tumor associated antigens (TAAs), hTERT has also been found frequently expressed in AML cells as well 26. However, the cytotoxicity of CTL to target cells depends on the effective recognition of pMHC complex. The downregulation of MHC expression and the lack of efficient antigen on tumor cells lead to the insufficiency of effective T cell activation, which is one of the important escaping mechanisms of tumor cells from immunotherapy. Hypomethylating agent treatment, including DAC treatment, is a standard care for AML patient 25. It has been reported that DAC treatment could induce the expression of CTAs and MHC in AML cells, but not in normal tissues 14, 15, 16. Thus, immunotherapy based on the DAC induced CTAs combined with DAC treatment could be a promising therapeutic strategy for AML patients. It could not only increase the targeting efficiency, but also inhibit the off-target effects. The latter topic should be investigated and confirmed, and it is under investigation in our lab.

In addition to the specificity of antigens proteins, one should keep in mind that MHC compatibility will also affect the efficiency of DC-CTL therapies. In our study, we selected NB4 cells, which express the HLA-A*1101 allele of MHC class I, the most common allele of MHC class I in China, to screen DAC treatment specific CTA, therefore the resulted CTAs should be more compatible to AML patients with HLA-A*1101 allele. This additional step may improve the potency of DC-CTL therapy by better binding of CTL to target cells.

Furthermore, antigen presentation by DC cells is also a key step that determines the efficiency of DC-CTL therapy's efficiency. The affinity of antigen peptide to MHC and the potency to be presented by DC cells are the prerequisite for successful antigen presentation. In our study, we deployed bioinformatic approaches to predict the binding affinity of peptides to MHC, and to evaluate the antigen epitope presentation potency, trying to find the most efficient CTA peptides for DC-CTL therapy. These bioinformatic analysis approaches could hugely improve the efficiency of antigen peptide finding.

Lastly, most current DC-CTL therapeutic strategies utilized unclarified mixture, such as AML cell lysate, which leads to the uncertainty of the effective targets, insufficiency of activated CTL cells, therefore instability of the treatment effects. In our study, purified antigen peptides were used in our study instead of crude lysate of tumor cells, which is one step further to achieve the certainty and the stability of DC-CTL immunotherapies.

However, based on the strategy we applied to investigate the potency of peptides, our study could not avoid certain limitations. One of such limitations was that we pooled all 4 different peptides to test their DC-CTL activation potency. Unavoidably, we could not find out the best peptide for each protein, which may limit the potency of the peptide stimulation and may introduce more off-target effect than single peptide. Our next step is to test the peptide potency individually to optimize the potency of the CTAp for DC-CTL immunotherapy.

## Figures and Tables

**Table 1:** selected peptides for MAGEA1 and hTERT after various bioinformatic analysis.

	CTA ID	HLA allele	CTA sequence	Antigen presentation potency	Binding affinity
NB4-MAGEA1					
	MAGEA1_NP_004979.3_9_95	HLA-A11:01	SLFRAVITK	0.8021	13
	MAGEA1_NP_004979.3_9_65	HLA-A11:01	TTINFTRQR	0.7029	65.3
	MAGEA1_NP_004979.3_10_95	HLA-A11:01	SLFRAVITKK	0.5501	29.9
	MAGEA1_NP_004979.3_9_269	HLA-A11:01	ALAETSYVK	0.6085	38.3
NB4-hTERT					
	hTERT_BAC11010.1_9_976	HLA-A11:01	ASLCYSILK	0.8747	5.8
	hTERT_BAC11010.1_10_1033	HLA-A11:01	RTAQTQLSRK	0.7134	49.2
	hTERT_BAC11010.1_9_561	HLA-A11:01	YVTETTFQK	0.7148	45
	hTERT_BAC11010.1_10_226	HLA-A11:01	SASRSLPLPK	0.5624	20.5

## Abbreviations

DC-CTL: dendritic Cell- Cytotoxic T Lymphocytes
AML: acute myeloid leukemia
DAC: decitabine
DNMT: DNA methyltransferases
CTA: cancer testis antigen
CTX: cyclophosphamide
MAGEA1: melanoma-associated antigen 1
hTERT: human telomerase reverse transcriptase
TAA: tumor associated antigens
TNF-α: tumor necrosis factor α
IFN-γ: interferon γ
CTX: cyclophosphamide
